# Evaluating If Children Can Use Simple Brain Computer Interfaces

**DOI:** 10.3389/fnhum.2019.00024

**Published:** 2019-02-04

**Authors:** Jack Zhang, Zeanna Jadavji, Ephrem Zewdie, Adam Kirton

**Affiliations:** ^1^Calgary Pediatric Stroke Program, Alberta Children’s Hospital Research Institute, University of Calgary, Calgary, AB, Canada; ^2^Hotchkiss Brain Institute, University of Calgary, Calgary, AB, Canada; ^3^Alberta Children’s Hospital Research Institute, University of Calgary, Calgary, AB, Canada

**Keywords:** brain computer interfaces (BCI), pediatrics, typically developing children, EEG, Emotiv EPOC

## Abstract

**Background:** The options for severely disabled children with intact cognition to interact with their environment are extremely limited. A brain computer interface (BCI) has the potential to allow such persons to gain meaningful function, communication, and independence. While the pediatric population might benefit most from BCI technology, research to date has been predominantly in adults.

**Methods:** In this prospective, cross-over study, we quantified the ability of healthy school-aged children to perform simple tasks using a basic, commercially available, EEG-based BCI. Typically developing children aged 6–18 years were recruited from the community. BCI training consisted of a brief set-up and EEG recording while performing specific tasks using an inexpensive, commercially available BCI system (EMOTIV EPOC). Two tasks were trained (driving a remote-control car and moving a computer cursor) each using two strategies (sensorimotor and visual imagery). Primary outcome was the kappa coefficient between requested and achieved performance. Effects of task, strategy, age, and learning were also explored.

**Results:** Twenty-six of thirty children completed the study (mean age 13.2 ± 3.6 years, 27% female). Tolerability was excellent with >90% reporting the experience as neutral or pleasant. Older children achieved performance comparable to adult studies, but younger age was associated with lesser though still good performance. The car task demonstrated higher performance compared to the cursor task (*p* = 0.027). Thought strategy was also associated with performance with visual imagery strategies outperforming sensorimotor approaches (*p* = 0.031).

**Conclusion:** Children can quickly achieve control and execute multiple tasks using simple EEG-based BCI systems. Performance depends on strategy, task and age. Such success in the developing brain mandates exploration of such practical systems in severely disabled children.

## Introduction

Brain computer interfaces (BCI), also known as brain machine interfaces, have the potential to improve the lives of people with severe disability. BCI typically work by detecting thought-induced changes in brain activity, relaying these signals to computer algorithms to detect associated patterns, and transmitting resulting commands to control effector devices such as a wheelchair, robotic arm, or computer mouse (Daly and Wolpaw, 2008). Original BCI systems were invasive, requiring implantation of recording devices directly into the brain, and ongoing physical external connections. However, the ability of non-invasive approaches, such as surface electroencephalogram (EEG)-based BCI, are rapidly expanding to accomplish similar utility (Wolpaw et al., 2002).

Few clinical circumstances are more tragic than locked-in syndrome where an intellectually capable individual is physically trapped in a body that does not move. This syndrome affects adults with conditions such as brainstem stroke, neuromuscular conditions like amyotrophic lateral sclerosis, and spinal cord injury where most BCI clinical evidence to date exists. In such circumstances, BCI provides opportunities for greater autonomy and interaction with the world by allowing individuals to directly connect their mental thoughts with BCI effector devices (Hochberg et al., 2012; De Vos et al., 2014). A young man with a cervical spinal cord injury and quadriplegia was recently able to reach out, grab a glass, and pour it using an implanted BCI linked to functional electrical stimulation of his arm (Bouton et al., 2016).

Despite the remarkable potential of BCI and the large global burden of severe disability in children, pediatric BCI studies have been limited (Breshears et al., 2011). Cerebral palsy is the leading cause of lifelong neurological disability, affecting 17 million people worldwide (Oskoui et al., 2013). Patients with severe quadriplegic cerebral palsy and intact cognition are ideal BCI candidates but few studies have focused on this population. Severe neuromuscular conditions and spinal cord injury provide additional pediatric examples of the locked-in syndrome. Children with perinatal stroke account for most hemiparetic cerebral palsy where motor issues are comparable to adult stroke hemiparesis (Kirton, 2013). BCI offer new avenues for rehabilitation such as recent adult stroke trials suggesting that combining BCI with traditional therapies may enhance function in hemiparetic patients (Ang et al., 2014; Donati et al., 2016). Limited studies have attempted to combine EEG-based BCI with functional electrical stimulation as a possible rehabilitation method for children with cerebral palsy (Jang et al., 2016; Kim and Lee, 2016). Fundamental to these potential pediatric applications is a gap in knowledge regarding if and how a developing, and often injured, young brain can acquire control of a BCI. An improved understanding of the abilities and challenges faced by children in using simple BCIs will inform protocol development for future studies in disabled pediatric populations.

Different mental strategies can be employed to drive BCI systems. For example, adults often use motor imagery (MI; Prasad et al., 2010; Ang et al., 2011). Imagined motor movements of the body generate reproducible alterations in sensorimotor rhythms such as EEG oscillations in the mu (7–13 Hz) and beta (13–30 Hz) bands (Nicolas-Alonso and Gomez-Gil, 2012). Evidence suggests that children have comparable sensorimotor rhythms related to hand movements (Kim et al., 2016). However, pediatric studies of BCI strategies are lacking and ability likely depends on cognitive capacity. For example, it may be non-intuitive for children to try and move an object using a common MI approach such as “think about squeezing both your hands.” A more practical approach might be using simpler “goal-oriented thoughts” such as imaging the desired effect, for example “think about the cursor or car moving toward the target.” In addition to strategy, the nature of the task attempted also influences BCI performance. Typical tasks, such as moving a cursor on a computer screen, are probably less interesting for children and the resulting lack of sustained engagement, attention, and motivation may be barriers to success. There is therefore a need to better understand pediatric specific issues of BCI applications.

We conducted a prospective, cross-over interventional study to estimate the ability of healthy school-aged children to perform simple tasks using different strategies and a basic, commercially available BCI. We hypothesized that older age and MI approaches would be associated with improved performance.

## Methods

### Participants

Participants were recruited from the Healthy Infants and Children’s Clinical Research Program (HICCUP ^1^), a population-based database of families interested in participating in medical research. Inclusion criteria were typical neurodevelopment, age 6–18 years, and informed consent/assent. Children with neurological or psychiatric conditions or taking neuroactive medication were excluded. Participants were compensated with two movie passes. Prior to participation, written assent and parental consent were obtained in accordance with the Conjoint Health Research Ethics Board, University of Calgary who approved the study. All subjects gave written informed consent in accordance with the Declaration of Helsinki.

### BCI System

**FIGURE 1:**
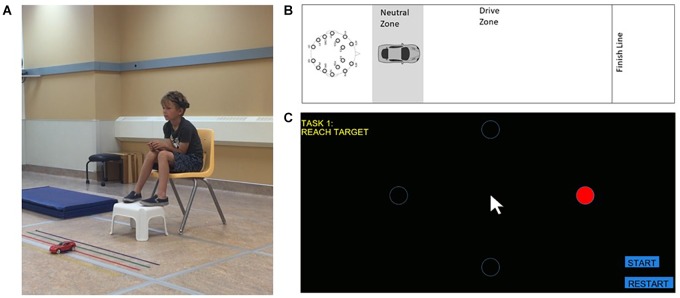
BCI system and tasks. **(A)** Child wearing EPOC headset completing the car task. Informed consent was obtained from the parent regarding publication. **(B)** Schematic of the car task. **(C)** Cursor task presented on a computer screen.

The EPOC (EMOTIV, United Sates), a commercially available 16-electrode dry contact headset, was used to collect and transmit EEG data. The head of each subject was measured and marked according to the standard 10–20 system. The headset had 14 electrodes located at AF3, AF4, F3, F4, F7, F8, FC5, FC6, P7, P8, T7, T8, O1, O2 and 2 additional reference electrodes on the right and left mastoids (Figure 1A). The headset electrodes were immersed in 0.9% saline to ensure a reliable connection before being placed on the head. Attempts to position the headset according to the 10–20 international system were made, however, the small head size of children and the structure of the device resulted in some variability in electrode placement (estimated within 1–2 cm from target location). The connection was verified using the EMOTIV Control Panel program (vendor provided software) which provides real-time, color-coded feedback on signal contact quality.

The EPOC had an internal sampling rate of 2048 Hz which was downsampled to 128Hz to produce a cleaner signal and wirelessly transmitted to a laptop computer. The control panel program acquired data and executed training. Training epochs were then defined by discrete 8-second samples of EEG data and stored accordingly. The classification depends on Event-Related Desynchronization phenomenon. It uses Probabilistic Neural Network (PNN) and Radial Basis Function (RBF) to distinguish between baseline and the training epochs to extract the relative signal indicative of the trained thought. Detection of such a trained action was then outputted as a virtual keystroke to be transmitted to one of the two effectors (car or cursor).

Upon arrival at the testing location, the participants and their parents read and signed the consent/assent forms. Participants were seated at a desk in a large room. They were assigned to 1 of 4 groups based on their order of entrance into the study. Participants attended two sessions, each separated by at least one week. In a single session, they completed both tasks using different strategies to prevent within-session learning effects. To compensate for order effects, the tasks were counterbalanced in the second session. Overall, participants were evenly split into 4 groups with initial training approach of car MI, car goal-oriented (GO), cursor MI, or cursor GO (Table 1).

**Table 1:** Sequence of task and strategy combinations for each group.

Groups	Session 1	Session 2
Car MI	Car MI → Cursor GO	Cursor MI → Car GO
Car GO	Car GO → Cursor MI	Cursor GO → Car MI
Cursor MI	Cursor MI → Car GO	Car MI → Cursor GO
Cursor GO	Cursor GO → Car MI	Car GO → Cursor MI


*MI: Motor imagery; GO: Goal-oriented thought*.

### Tasks and Strategies

Over two sessions, participants attempted a remote-controlled car and computer cursor task, using two different thought strategies, GO and MI. Participants attempted both tasks in each session using a randomly assigned thought strategy in random order. Order of task completion and strategies used were alternated for the second session.

In each task, participants were required to prevent the car or cursor from moving for a given period of time and to then use a preassigned thought strategy to move the object to its target. The car moved in a forward direction to cross a finish line (Figure 1B), while the cursor moved in a rightward direction to a circular target on a computer screen (Figure 1C). Both tasks were designed such that they could be completed in comparable time.

Participants began each session by training a neutral and movement command. During neutral training, participants were asked to count down backward from 10 in order to engage in a task that is not associated with the car or cursor moving task. Participants were randomly assigned to use one of two thought strategies during the movement training. If assigned to MI, participants were asked to imagine opening and closing both of their hands to move the car or cursor during the tasks. For the GO strategy, participants were asked to visualize the object of interest moving toward a designated target. Neutral and movement commands were trained together in an ordered sequence of doubles; two neutral trainings were accompanied by two movement trainings which was repeated four times. In total, each command was presented 8 times. Immediately following this was the testing phase in which participants completed 10 trials of the task.

Each trial of the testing phase consisted of a neutral and movement period. During neutral, participants were asked to recount their previous training to keep the car or cursor idle for 5 s. They were then instructed to use their designated thought strategy to move the car or cursor to its target within 20 s. After completing the first task, participants immediately trained for the second task. Event markers were manually added during the training phase to define the timing of all events.

Object type and the direction of movement varied between both tasks. The car task was assumed to be more engaging as it involved the movement of a physical object accompanied by auditory feedback provided by the electric motor; only visual feedback was provided in the cursor task. The direction of object movement was different for both tasks in order to prevent possible learning effects with the GO command. All trials were video recorded or data logged for offline analysis and validation.

The primary outcome measure was success of task completion quantified by Cohen’s kappa, a measure of agreement between the commands issued by the investigator and the actions performed by the participant. This is consistent with multiple adult BCI training studies (Daly et al., 2013; Scherer et al., 2013) facilitating comparison of our results. A score of 1 represented perfect agreement and a score of 0 represented chance agreement. In each trial, to correctly follow the neutral command, participants needed to hold in the neutral state for the entire 5 s. To correctly follow the movement command, participants needed to cause at least some movement, regardless of distance, in the first 8 s of the 20 s trial. The difference in timing is due to the increased difficulty of the neutral phase.

### BCI Experience Survey

Participants answered a series of questions assessing their personal characteristics, mental tiredness, perceived enjoyment, difficulty, and preferences on a 5-point Likert scale. Any potential side effects, adverse events, or unpleasant features were also recorded.

### Analysis

Cohen’s kappa, the primary outcome measure of BCI skill, was tested for normality. A two-way repeated measures ANOVA compared the impact of task and strategy on Cohen’s kappa. Missing data was removed for repeated measures ANOVA. The impact of age was examined with a Pearson correlation between individual mean Cohen’s kappa from all four learning attempts. The between-session and within-session learning effects were examined with a two-tailed paired *t*-test. Changes between trials were analyzed with a two-way repeated measures ANOVA. The specific impact of strategy on trial-by-trial learning was examined with a Pearson correlation. All data were analyzed using SPSS 24. Graphs were created using GraphPad 5.

## Results

### Population

We recruited 30 healthy children of whom 26 completed the entire study (73% male, mean [SD] 13.2 [3.6] years, range 6–18). Three participants were unable to be scheduled for the second session and one dropped out as they found the headset uncomfortable. Most children enjoyed participating with 55% reporting a positive experience (4 or 5 on a 5-point Likert scale) and only 1 (4%) reporting a negative experience. All sessions were less than 1 h with an average of 45 min. Half of the participants reported mental fatigue by the end of the session, typically during the final 10 min. Among those reporting a negative or neutral experience, the most common complaint (50%) was that the headset applied too much pressure and caused discomfort. No other adverse events were reported.

### BCI Performance

**FIGURE 2:**
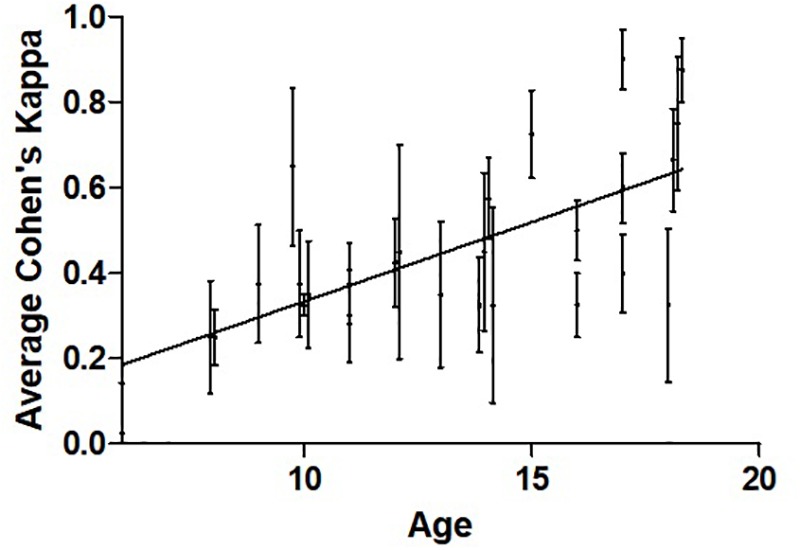
Relationship between participant average Cohen’s kappa and age. Pearson’s *r* = 0.661, *n* = 26, *p* < 0.001. The mean and variance across four training sessions are shown for each participant. The data points are slightly shifted to reveal vertical error bars which represent SEM.

Across the entire population and all tasks, the primary outcome was normally distributed with an average Kappa score of 0.46 [0.21] and range of 0.025–0.90. Performance correlated with increasing age (Figure 2, Pearson’s *r* = 0.632, 95%CI 0.37–0.83, *p* < 0.001). Sex was not associated with performance. Overall, 11/26 (42%) participants achieved a Cohen’s kappa of 0.4 or higher, a cut-off for competency often used in adult BCI studies (Scherer et al., 2013; Jeunet et al., 2016).

### Task and Strategy

**FIGURE 3:**
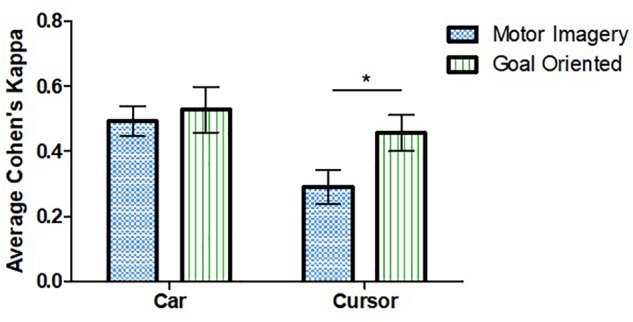
Effect of strategy and task on average Cohen’s kappa. Performance was higher for the car task compared to cursor. Goal-oriented approaches were more successful for the cursor task. ^∗^Represent statistically significant difference with *p* < 0.05. Error bars represent SEM.

The repeated measures ANOVA demonstrated an effect of both task and strategy (Figure 3). Higher mean performance scores were observed for the car (0.518 [0.295]) as compared to the cursor task (0.393 [0.294], *F*(1, 25) = 5.52, *p* = 0.027. Effect of strategy was also significant as the mean Cohen’s kappa for the GO strategy (0.506 [0.315]) was higher than for MI (0.403 [0.277], *F*(1, 25) = 5.20, *p* = 0.031).

Tukey’s *post hoc* test examined the effect of strategy for each task individually. A difference was observed for the cursor task in which the GO approach (0.469 [0.280]) was more successful than MI (0.318 [0.294], *t*(25) = 2.602, *p* = 0.015). No such difference was observed for the car task. The post-experiment survey revealed no differences in personal preference for one strategy over the other: 41% preferred GO, 45% preferred MI. There was no significant interaction between task and strategy, *F*(1, 25) = 2.817, *p* = 0.106.

**FIGURE 4:**
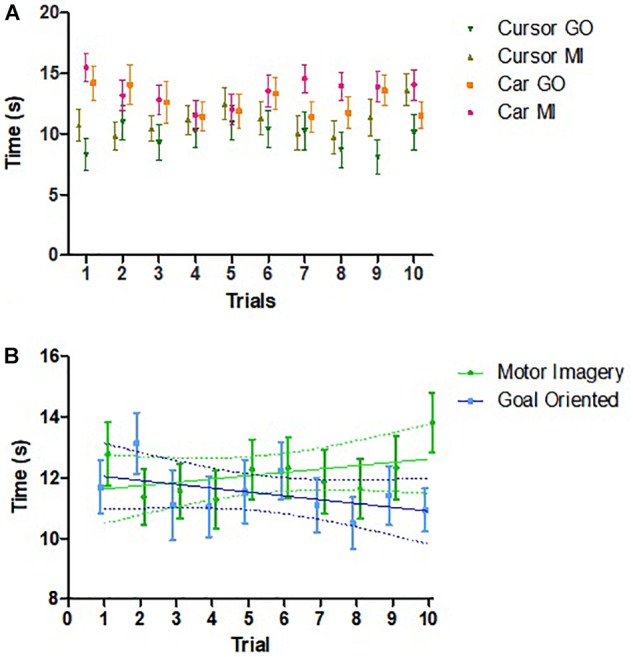
Changes in performance across trials. **(A)** Comparison of the changes in time among different groups across trials (*n* = 26). No effect of trial was found. **(B)** Relationship between time required to complete session and trial number by strategy (*n* = 26). For MI strategy, Pearson’s *r* = 0.435, *p* = 0.209. For goal oriented strategy, Pearson’s *r* = –0.513, *p* = 0.130. Error bars represent SEM. Points are slightly shifted for clarity.

Comparison within subjects of performance between the first (0.425 [0.226]) and second (0.488 [0.243]) sessions revealed possible improved performance in the second session, *t*(25) = 1.722, 95% CI –0.14 to 0.01, *p* = 0.097. Across all 52 sessions, there were no within-session learning effects, *t*(51) = 0.955, 95% CI –0.06 to 0.16, *p* = 0.344. Performance over time across the 10 trials demonstrated no effect of trial number on time, *F*(9, 106) = 1.03, *p* = 0.415 (Figure 4A). There was also no association between strategy and learning across trials (Figure 4B).

## Discussion

Our study demonstrates that children can use a simple, commercially available BCI and different mental strategies to train basic tasks in minimal time. Age, task, and strategy appear to influence the ability of children to use such BCI systems. The combination of significant efficacy, ease of application, low cost, and favorable tolerability suggests high potential for non-invasive BCI systems in pediatric clinical populations.

To our knowledge, there have been no prior studies quantifying BCI skills in children using such simple BCI systems. Consistent with BCI studies to date, success rates appear highly individualized. Adult BCI studies typically label 15–30% of participants as BCI “illiterate” based on a cut-off of 70% classification accuracy or 0.40 on Cohen’s kappa (Scherer et al., 2013; Jeunet et al., 2016). Among our 26 participants, nearly half achieved such proficiency with much higher rates observed in some individuals. Our higher BCI illiteracy could be due to children 10 years of age or younger having more difficulties (Figure 2). This does not resolve whether adults are superior to children in BCI performance but supports the ability of young people to control BCI systems at a practical level.

BCI performance in children was higher on the car task compared to the cursor task. We hypothesized this due to greater engagement in moving a flashy, red toy as compared to the more mundane cursor. Although we did not fully quantify task interest, an important aspect to controlling BCI is maintaining attention (Nijboer et al., 2010). Such abilities are also developmentally sensitive and may relate to our observations of lower performance in younger participants. Our results suggest that the task is an important consideration and should aim to be captivating when intended for pediatric populations. Other factors, such as gaming applications or peer competition might further motivate children.

The GO strategy was associated with better performance as compared to MI which was unexpected. While MI is the most commonly used BCI mental task (Scherer et al., 2013), it might be less intuitive in some cases. The main effect of strategy primarily stemmed from a decrease in MI performance during the cursor task as there was no difference between performance using the two strategies for the car task (Figure 3). One possible explanation for our result is that the cursor task moved the cursor to the right while the car task moved the car forward. Moving an object to the right by opening and closing both hands seem unintuitive.

We anticipated that children would improve their performance with practice, but only found a non-significant difference. Furthermore, a possible association between time and trial number emerged when strategies were examined separately (Figure 4B). One possible explanation could be the mental fatigue experienced toward the end of the trial. Another possibility could be that children had difficulties reproducing the same thought strategy throughout the 10 trials. Studies including more training trials or sessions may be able to better elucidate learning effects of BCI performance in children.

Our results carry translational significance. Cerebral palsy affects 17 million people worldwide, a large portion of whom have severe quadriplegia with minimal or no motor function. Within this group, a significant proportion have preserved cognition, resulting in a locked-in state. There is only limited evidence that pediatric cerebral palsy populations can benefit from BCI (Jang et al., 2016; Kim and Lee, 2016). While implantable BCI is accomplishing remarkable functional achievements in adults (Bouton et al., 2016), such invasive systems are neither available nor practical in young children. Instead, working backward from the simple technologies we studied here may allow pediatric BCI applications to achieve more functionality in disabled pediatric populations, allowing them to interact with their world and achieve greater levels of independence and quality of life.

### Limitations

Differences between our BCI skill measures and previous research (Pfurtscheller and Neuper, 2001) limits comparisons. This is due to the practical requirement of our BCI system for an all-or-none measurement. To better compare children and adults, future trials might test both populations on identical protocols. Although the systems employed carried multiple compensations for potential artifacts such as movement, we did not have access to confirm their effectiveness. We have not yet studied the origin of the EEG signals driving BCI success where the use of richer EEG-BCI systems might further inform pediatric differences though invasive cerebral monitoring suggests mechanisms are likely similar (Breshears et al., 2011). There was substantial variability in individual’s BCI performance (Figure 2), suggesting larger sample sizes may be required to detect more subtle differences. Similarly, lengthening the testing phase to include more trials could better define the effects of age, sex, strategy, and learning. Another possible issue of generalizability relates to selection bias. Our population was taken from families who have demonstrated interest in research with potential implications on levels of motivation, intelligence or other factors which may not be generalizable to all pediatric populations.

## Conclusion

Children can quickly achieve control and execute multiple tasks using simple EEG-based BCI systems. Performance depends on strategy, task and age. Such success in the developing brain mandates exploration of such practical systems in severely disabled children.

## Author Contributions

JZ conceptualized and designed the study, collected the data, conducted analyses, and drafted and revised the initial manuscript. ZJ collected the data and reviewed and revised the manuscript. EZ and AK conceptualized and designed the study, and reviewed and revised the manuscript.

## Conflict of Interest Statement

The authors declare that the research was conducted in the absence of any commercial or financial relationships that could be construed as a potential conflict of interest.

## Footnotes

1 www.hiccupkids.ca

## Abbreviations

BCI: brain computer interface
ECP: Emotive control panel
GO: goal oriented
MI: motor imagery
RC: remote controlled
